# The divergence of alternative splicing between ohnologs in teleost fishes

**DOI:** 10.1186/s12862-021-01833-6

**Published:** 2021-05-25

**Authors:** Yuwei Wang, Baocheng Guo

**Affiliations:** 1 Key Laboratory of Zoological Systematics and Evolution, Institute of Zoology, Chinese Academy of Sciences, Beijing, 100101 China; 2 University of Chinese Academy of Sciences, Beijing, 100049 China; 3 Center for Excellence in Animal Evolution and Genetics, Chinese Academy of Sciences, Kunming, 650201 China

**Keywords:** Gene duplication, Alternative splicing, Ohnolog, Singleton, Teleost

## Abstract

**Background:**

Gene duplication and alternative splicing (AS) are two distinct mechanisms generating new materials for genetic innovations. The evolutionary link between gene duplication and AS is still controversial, due to utilizing duplicates from inconsistent ages of duplication events in earlier studies. With the aid of RNA-seq data, we explored evolutionary scenario of AS divergence between duplicates with ohnologs that resulted from the teleost genome duplication event in zebrafish, medaka, and stickleback.

**Results:**

Ohnologs in zebrafish have fewer AS forms compared to their singleton orthologs, supporting the function-sharing model of AS divergence between duplicates. Ohnologs in stickleback have more AS forms compared to their singleton orthologs, which supports the accelerated model of AS divergence between duplicates. The evolution of AS in ohnologs in medaka supports a combined scenario of the function-sharing and the accelerated model of AS divergence between duplicates. We also found a small number of ohnolog pairs in each of the three teleosts showed significantly asymmetric AS divergence. For example, the well-known ovary-factor gene *cyp19a1a* has no AS form but its ohnolog *cyp19a1b* has multiple AS forms in medaka, suggesting that functional divergence between duplicates might have result from AS divergence.

**Conclusions:**

We found that a combined scenario of function-sharing and accelerated models for AS evolution in ohnologs in teleosts and rule out the independent model that assumes a lack of correlation between gene duplication and AS. Our study thus provided insights into the link between gene duplication and AS in general and ohnolog divergence in teleosts from AS perspective in particular.

**Supplementary Information:**

The online version contains supplementary material available at 10.1186/s12862-021-01833-6.

## Background

Gene duplication is a common phenomenon in genome, and is deeply believed to play important roles in organismal evolution [1]. Gene duplication could result from unequal crossing over [2], retroposition [3], and whole genome duplication (WGD) [4, 5]. Evolutionary fates of duplicated genes, nonfunctionalization [1], subfunctionalization [6, 7], neofunctionalization [1], and sub-neofunctionalization [8], have been well known in the past two decades [9], with extensive studies of divergence between duplicates in many aspects, e.g., sequence, expression, and protein interaction [10–12]. However, functional innovation in duplicates and its significance in evolution continues to be astonishing, e.g., in human brain size expansion [13–15] and origin of the bulbus arteriosus in teleosts [16]. It says that our understanding of divergence between duplicates and their evolutionary significance is far from complete, which might be particularly relevant in non-human organisms.

Alternative splicing (AS), the production of different mature transcripts from the same primary RNA sequence, is a post-transcriptional process that allows a single gene to encode multiple proteins by including or excluding certain exon from the mature mRNA [17]. AS is a common phenomenon in eukaryotes, which greatly increases gene complexity at protein level [18, 19]. For example, ~ 95% human multiple-exon genes show alternative splicing [20]. Interestingly, multiple-exon genes tend to be retained long after duplication in various organisms [21, 22]. Thus, it would be interesting to know the divergence of AS between duplicates.

Earlier studies suggest there is link between gene duplication and AS in evolution. Three models for the evolution of AS between duplicates have been proposed, including the independent model, where no correlation between gene duplication and AS, the function-sharing model, where duplicates reciprocally retain AS forms in their ancestor, the accelerated model, where both duplicates evolve more AS forms compared to their ancestral gene [23, 24]. Su et al. [25] proposed that the function sharing model was the main model of AS evolution after gene duplication and found AS was preferentially lost in young duplicates and new AS form is acquired in old duplicates. Abascal et al. [26] found the divergence of AS between duplicates follows the sharing model in fish genomes. Kopelman et al. [27] found an inverse correlation between the size of a gene’s family and its use of alternatively spliced isoforms in human and mouse and Su et al. [25] confirmed this finding, suggesting gene duplication and AS rates are not independent evolutionary properties of a gene. Talavera et al. [28] found that the amounts of AS and duplication per gene were anticorrelated even when accounting for different gene functions or sequence divergence. However, the reverse correlation between level of AS and family size is controversial [29]. Although those findings have scientifically advanced our understanding relationships between gene duplication and AS, earlier studies usually took family size as measurement of gene duplication with focus on human and mouse, and genome-wide study in non-human organisms which have been experienced WGD and contain many ohnologs in their genomes is rare.

WGD plays an important role in new function involving in genomes and promotes species diversification [30]. Teleost fishes are the most species-rich group of extant vertebrates. A round of WGD, the teleost genome duplication (TGD), occurred in ancestor of teleosts [31, 32]. Thus, thousands of ohnologs—duplicates originating from WGD exist in teleost genomes, providing the best opportunity for studying the divergence of alternative splicing between duplicates long after duplication. To better explore the divergence of alternative splicing between duplicates, we characterized alternative splicing forms in both singletons and duplicates in genomes of three teleost fishes, zebrafish (*Danio rerio*), medaka (*Oryzias latipes*), and stickleback (*Gasterosteus aculeatus*), with aid of comprehensive RNA-seq data.

## Results

### Transcript number difference between ohnologs in the three teleost fishes

;Ohnologs (referred to as 1to2 genes) that resulted from TGD and singletons (referred to as 1to1 genes) were retrieved from Inoue et al. [33] (Additional file 1: Table S1). Only exact 1to2 and 1to1 genes were used in following analyses to avoid false positive gene identification [21]. The number of singletons and ohnolog pairs used in each of the three teleost species and their mean transcript number (the number of transcripts for each gene in Ensembl) are listed in Table 1. The median transcript number of both singletons and ohnolog is 2 in zebrafish, and 1 in both medaka and stickleback. The transcript number of ohnologs (mean of 2.22 ± 0.04) is significantly larger than that of singletons (mean of 2.05 ± 0.02) in zebrafish (Wilcoxon rank-sum test, *P* = 5.00 ⋅ 10^− 5^), and no difference in medaka or stickleback (Wilcoxon rank-sum tests, *P* > 0.61). Next, we compared transcript number between ohnologs and their singleton orthologs cross species by assigning ohnolog pairs to two random groups in each species (Additional file 2: Fig. S1; Additional file 1: Table S2). In zebrafish, transcript number in ohnologs is significantly more than that in their singleton orthologs in both medaka and stickleback (Wilcoxon signed-sum tests, *P* < 0.01; Additional file 2: Fig. S1). In medaka, transcript number in ohnologs is significantly less than that in their singleton orthologs in zebrafish (Wilcoxon signed-sum tests, *P* < 0.01; Additional file 2: Fig. S1), and not significantly less than that in their singleton orthologs in stickleback (Wilcoxon signed-sum tests, *P* > 0.01; Additional file 2: Fig. S1). In stickleback, transcript number in ohnologs is significantly less than that in their singleton orthologs in zebrafish (Wilcoxon signed-sum tests, *P* < 0.01; Additional file 2: Fig. S1), and has no difference from that in singleton orthologs in medaka (Wilcoxon signed-sum tests, *P* > 0.01; Additional file 2: Fig. S1).

**Table 1:** Numbers of singletons and ohnologs and numbers of their transcripts in Ensembl and predicted alternative splicing (AS) forms with RNA-seq data

	Singletons		Ohnologs	
	Gene number	Transcript number	Gene pair	Transcript number
Zebrafish	3792	2.05 ± 0.02	581	2.22 ± 0.04
Medaka	3362	1.33 ± 0.01	497	1.31 ± 0.02
Stickleback	3622	1.37 ± 0.01	548	1.39 ± 0.02

	Gene number	AS number	Gene pair	AS number
Zebrafish	3777	4.47 ± 0.07	578	4.79 ± 0.13
Medaka	3349	6.37 ± 0.10	492	6.23 ± 0.18
Stickleback	3598	5.96 ± 0.09	542	6.74 ± 0.20

### AS number difference between ohnologs in the three teleost fishes

Mean AS forms in singletons and ohnologs based on RNA-seq data in each of the three teleost species are listed in Table 1, where single exon genes are excluded from either singletons or ohnologs. We first found that both singletons and ohnologs with more exons tend to have more AS forms (Additional file 3: Fig. S2). We then compared AS forms between singletons and ohnologs within each teleost. AS forms are not significantly different between ohnologs and singletons in either zebrafish or medaka (Wilcoxon rank-sum tests, *P* > 0.11), but in stickleback, ohnologs have significantly more AS forms (mean of 6.74 ± 0.20) than singletons (mean of 5.96 ± 0.09) (Wilcoxon rank-sum test, *P* = 2.76 ⋅ 10^− 3^).

**Fig. 1:**
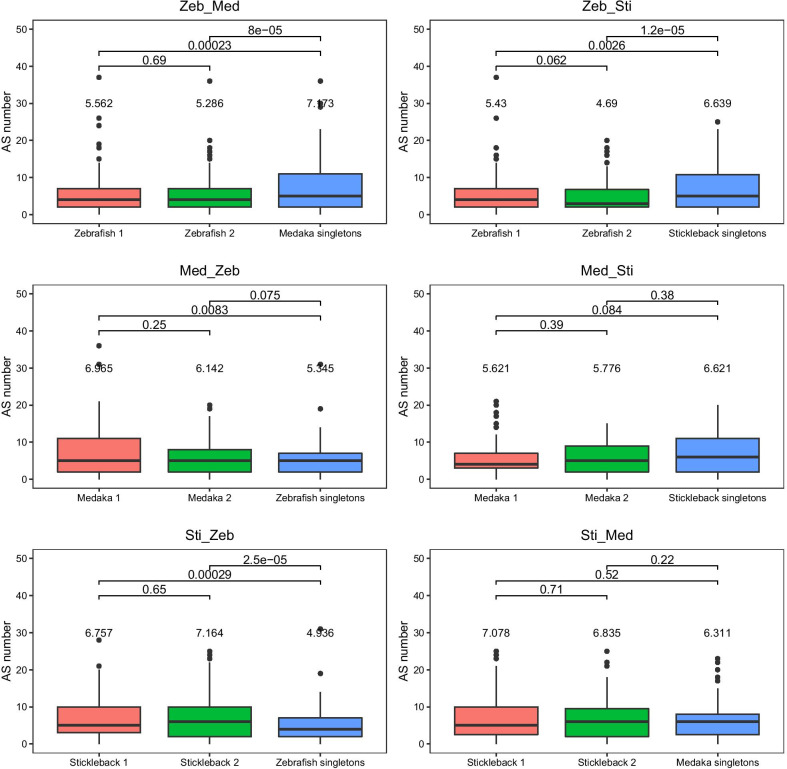
Alternative splicing forms between ohnologs and their singleton orthologs. The number on the top of the box is the mean of each group

Next, we compared AS forms between ohnologs and their singleton orthologs cross species by assigning ohnolog pairs to two random groups in each species (Fig. 1; Additional file 1: Table S2). In zebrafish, AS forms in ohnologs are significantly less than that in their singleton orthologs in both medaka and stickleback (Wilcoxon signed-sum tests, *P* < 0.01; Fig. 1). In medaka, AS forms in ohnologs are more that in their singleton orthologs in zebrafish, in which is only statistically significant in one comparison (Wilcoxon signed-sum tests, *P* < 0.01; Fig. 1); AS forms in ohnologs are less than that in their singleton orthologs in stickleback, in which no significant difference is found (Wilcoxon signed-sum tests, *P* > 0.01; Fig. 1). In stickleback, AS forms in ohnologs are significantly more than that in their singleton orthologs in zebrafish (Wilcoxon signed-sum tests, *P* < 0.01; Fig. 1), and are more than that in their singleton orthologs in medaka (Wilcoxon signed-sum tests, *P* > 0.01; Fig. 1).

**Fig. 2:**
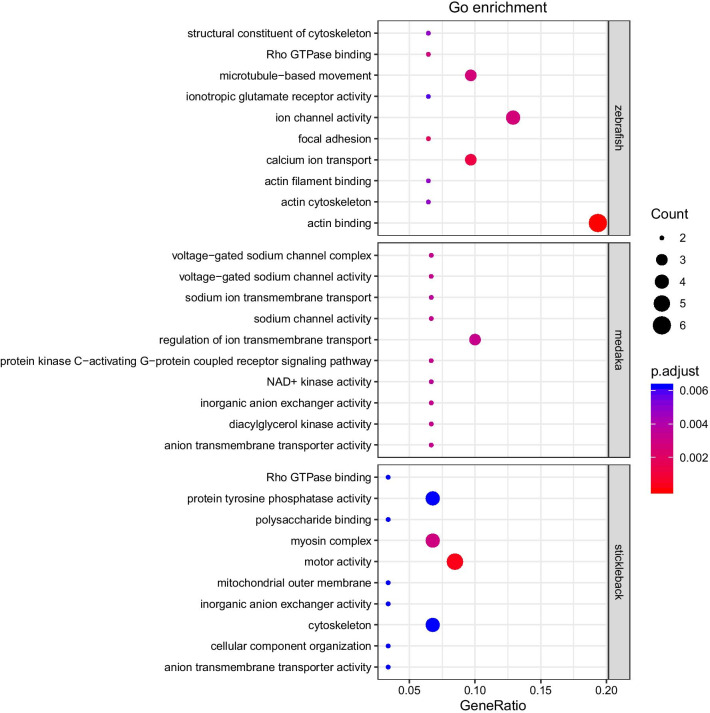
Significantly enriched GO terms of ohnologs with asymmetric alternative splicing forms in zebrafish, medaka, and sticklebacks

Finally, a small number of ohnolog pairs have significantly asymmetric AS forms, i.e., 16 (2.77%) in zebrafish, 17 (3.46%) in medaka, and 33 (6.09%) in stickleback (exact binomial test, FDR adjusted *q* < 0.05; Additional file 1: Table S1). These ohnologs are significantly enriched in GO terms, e.g., actin binding in zebrafish, regulation of ion transmembrane transport in medaka, and motor activity in stickleback (Fig. 2). GO-like enrichment of anatomical terms analysis shows that expression of these ohnologs is preferentially found in several neural tissues, i.e. anterior lateral line system, hindbrain, dorso-rostral cluster, midbrain, ventral part of telencephalon, and ventro-rostral cluster (Table 2, Additional file 1: Table S3).

**Table 2:** GO-like enrichment of anatomical terms analysis (FDR adjust *q* < 0.05) of ohologs with significantly asymmetric splicing events in zebrafish using BgeeDB (https://bgee.org/)

Organ ID	Organ name	*P* value	FDR adjust *q*	Genes
UBERON:2,001,468	Anterior lateral line system	0.0001	0.041	ENSDARG00000020581 ENSDARG00000037496 ENSDARG00000059368
UBERON:0002028	Hindbrain	0.0001	0.041	ENSDARG00000023542 ENSDARG00000030832 ENSDARG00000037496 ENSDARG00000040110 ENSDARG00000055754 ENSDARG00000058203 ENSDARG00000059368 ENSDARG00000060123
UBERON:2,007,001	Dorso-rostral cluster	0.0002	0.041	ENSDARG00000037496 ENSDARG00000059368
UBERON:0001891	Midbrain	0.0002	0.041	ENSDARG00000023542 ENSDARG00000030832 ENSDARG00000037496 ENSDARG00000040110 ENSDARG00000055754 ENSDARG00000058203 ENSDARG00000059368 ENSDARG00000060123
UBERON:0000204	Ventral part of telencephalon	0.0002	0.041	ENSDARG00000023542 ENSDARG00000037496 ENSDARG00000059368
UBERON:2,007,002	Ventro-rostral cluster	0.0002	0.045	ENSDARG00000037496 ENSDARG00000059368

## Discussion

In this study, we explored the divergence of AS between ohnologs in three well studied teleosts with both gene annotation in database and RNA-seq data. In the following, we discussed our results in relation to evolutionary relationships between gene duplication and AS in general and evolutionary significance of ohnolog divergence in teleosts from AS perspective in particular.

### AS divergence in ohnologs in teleosts

Being two distinct sources of evolutionary innovation in protein diversification, the evolutionary link between gene duplication and AS has been studied at gene level since the early 2000 s [34]. Genome-wide studies suggested gene duplication and AS are inversely correlated evolutionary mechanisms, e.g., duplicates having fewer alternative splicing forms than singletons [25, 27]. Roux and Robinson-Rechavi [29] argued that those findings by Kopelman et al. [27] and Su et al. [25] no longer hold true when taking evolutionary time into account carefully. As such, Chen et al. [35] found the amounts of AS and duplication were positively correlated in ancient duplications events. Three models, the independent model, the function sharing model, and the accelerated model are proposed to explain AS evolution after duplication by comparing the number of AS forms between duplicates and singletons [23, 24]. We tackled the evolutionary link between gene duplication and AS using ohnologs that were generated by the TGD at same time in zebrafish, medaka, and stickleback. We first compared AS forms in ohnologs and singletons within each species. We found that in terms of average value, both gene annotation in public database and AS prediction based on RNA-seq data show that AS forms in ohnologs are close to those in singletons in zebrafish and medaka, and are more than those in singletons in stickleback (Table 1). However, gene annotation in public database considerably underestimates AS forms in teleost genes compared to prediction with RNA-seq data, and could not fairly demonstrate AS evolution in ohnologs. Thus, we utilize results of RNA-seq data to understand AS evolution in ohnologs. Next, we decipher the evolution of AS after duplication by comprising the number of AS forms between ohnologs and their singleton orthologs cross species. We found that the evolutionary link between gene duplication and AS in each of the three teleosts supports different models proposed by Reddy et al. [24]. In zebrafish, number of AS forms in ohnologs is less than that in their singleton orthologs, supporting the function sharing model in which each copy of duplicates retain partial number of AS forms in their ancestor and the number of AS forms in duplicate gene is reduced compared to their singleton orthologs [24]. In stickleback, number of AS forms in ohnologs is more than that in their singleton orthologs, supporting the accelerated model in which the number of AS forms is increased in each copy of duplicates [24]. In Medaka, the number of AS forms in part of ohnologs is more than that in their singleton orthologs and in part of ohnologs less than that in their singleton orthologs, supporting both the accelerated model and the function sharing model. All results in the three teleosts support evolutionary link between gene duplication and AS and rule out the independent model that assumes a lack of correlation between gene duplication and AS and the number of AS forms in duplicates is similar to that in their singleton orthologs [24]. Our results thus suggest a combined scenario of function-sharing and accelerated models for AS evolution in ohnologs, suggesting both subfunctionalization and neofunctionalization occurred in ohnologs that have been long retained after WGD by AS form loss and gain [25]. This is understandable from the perspective of selection pressure change after duplication. Both duplicates typically experience relaxed purifying selection [6, 7], which allows for reciprocal AS loss in duplicates in the functional sharing model and for AS gain in duplicates in the accelerated model. Additionally, it is also not surprised that the AS divergence model in ohnologs is species-specific in the three studied teleosts, considering the profile of ohnologs retained in teleost genomes after TGD is species-specific.

However, two methodological aspects relating to interpretation of observations abovementioned deserve to be discussed. First, we notice that in disentangling models of AS divergence in ohnologs, we comprised the number of AS forms between ohnologs and their singleton orthologs cross species. However, those singletons we used might be not ideal proxies, given that they have gone through their own evolutionary history in which AS gain and loss occurred. It says that the models of AS divergence in ohnologs could be ideally studied in species that was experienced WGD recently and also had closely related outgroup that escapes from WGD. Second, considering the widespread tissue-specific gene expression, the distinct divergence pattern of AS in ohnologs among the three teleosts studies we observed might result from pooling unequal amount of RNA-seq data from multiple tissues (Additional file 1: Table S4). We thus investigated AS divergence in ohnologs with equal amont of RNA-seq data from liver in which comprehensive RNA-seq data is available for AS predication in each of the three teleosts tissues (Additional file 1: Table S4). It is not surprisingly that the number AS from RNA-seq data in liver only is less than that from pooled RNA-seq data in multiple tissues, but the divergence pattern of AS in ohnologs from RNA-seq data in liver only is similar to that in multiple tissues in each of the three teleosts (Additional file 4: Fig. S3). It says that our observation of the distinct AS divergence pattern in ohnologs among the three teleosts studies is unlikely affected by using pooling unequal amount of RNA-seq data from multiple tissues.

### Evolutionary significance of AS divergence in ohnologs in teleosts

**Fig. 3:**
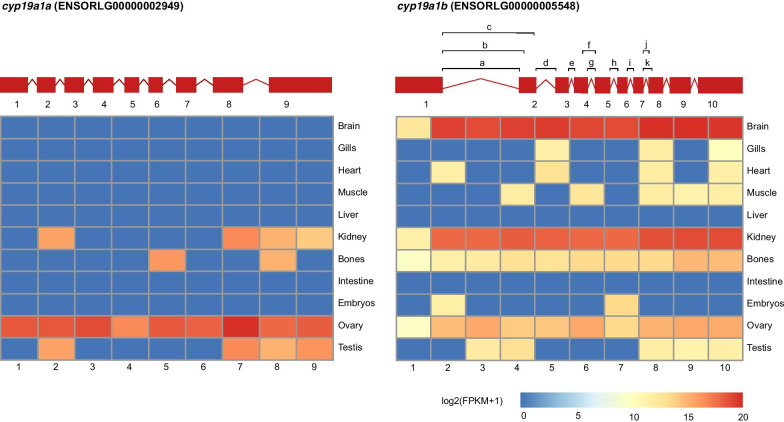
The alternative splicing graph for *cyp19a1a* and *cyp19a1b* and their expression profile in medaka. Eleven predicted alternative splicing events in *cyp19a1b* are labeled as **a**–**k**. **a–c** are A3SS (Alternative 3′ Splice Site) type of alternative splicing events; **d**, **e**, **g**, **h**, **i**, and **j** are RI (Retained Intron) type of alternative splicing events; f is RI or A5SS (Alternative 5′ Splice Site) type of alternative splicing events; k is RI or A3SS type of alternative splicing events, according to Goldstein et al. [44]. Heatmaps are based on exon expression on a log2(FPKM + 1) scale

WGD events have been deeply believed to shape the history of many evolutionary lineages, especially in teleosts. Reciprocal loss of ohnologs in different teleost lineages after TGD might have contributed to teleost diversification [36]. Lineage-specific re-diploidization of ohnologs could last over tens of millions of years and is assumed to be responsible for specific adaptations and diversification in salmons that underwent salmonid-specific WGD ~ 95 MYA [37]. It says that WGD provided teleosts with diversification potential that can become effective much later, such as during phases of environmental change, by generating thousands of ohnologs [33, 37, 38]. Sub/neofunctionalization of an ohnolog—*elastin* gene generated by TGD contributes to origin of the bulbus arteriosus, an evolutionarily novel organ in teleost heart outflow tract [16]. Glasauer and Neuhauss [38] summarized evolutionary consequences of ohnologs in teleosts after TGD from various perspectives. Interestingly, a few studies dedicate effort to explore genome-wide divergence pattern of alternative splicing in ohnologs in teleosts [39], although pufferfish (*Takifugu rubripes*) has served the very first case of subfunctionalization in ohnologs from AS divergence perspective [34]. It might be due to insufficient gene annotation in non-human genomes in general, for example, transcript number of genes in teleosts is significantly fewer than that of their human orthologs (Wilcoxon signed-rank tests, *P* < 2.2 ⋅ 10^− 16^; Additional file 1: Table S1) in current genomic database. The rapid accumulation of next generation sequencing data allows us to explore ohnolog divergence in teleosts from AS perspective. As such, we show that AS significantly diverges in ohnologs in teleosts as well as sequence, expression, and protein interaction divergence [38]. For example, a small number of ohnolog pairs show significantly asymmetric AS divergence in each of the three studied teleosts, which might suggest functional divergence between ohnologs. An ohnolog pair of aromatase genes in medaka, *cyp19a1a* (ENSORLG00000002949) and *cyp19a1b* (ENSORLG00000005548), shows significantly asymmetric AS divergence based on RNA-seq data, with no AS form being found in *cyp19a1a* but 11 AS forms in *cyp19a1b*. *cyp19a1* is considered the most conserved ovary-factor in vertebrates and expressed in various tissues with multiple AS forms [40]. Earlier in teleosts, *cyp19a1a* and *cyp19a1b* are found to be expressed in ovaries and the brain, respectively [40]. However, it shows that both *cyp19a1a* and *cyp19a1b* are actually expressed in multiple tissues in teleosts [41, 42], which is also confirmed with RNA-seq data in this study (Fig. 3). Domingos et al. [42] found that *cyp19a1a* was expressed in testes in levels similar to, or higher than those in ovaries in barramundi but its full coding sequence was absent in the males due to exon splicing. Taken those studies together, it suggests that functional divergence between *cyp19a1a* and *cyp19a1b* has been accompanied by asymmetric alternative splicing divergence in teleosts. Considering the amount of ohnologs in teleost genomes [33] and the unneglected fraction of them with significantly asymmetric AS divergence, our study thus from the perspective of alternative splicing divergence in ohnologs shows that the TGD increased the genomic complexity of teleost.

## Conclusions

In conclusion, we characterized alternative splicing divergence between ohnologs that resulted from TGD in three teleost genomes with the aid of RNA-seq data. We found that alternative splicing evolution in ohnologs supported a combined scenario of function-sharing and accelerated models and ruled out the independent model that assumed a lack of correlation between gene duplication and alternative splicing. A small number of ohnolog pairs showed significantly asymmetric alternative splicing divergence, which might result in functional divergence between duplicates. Taken together, our study provided insights into the link between alternative splicing and gene duplication in general and ohnolog divergence in teleosts from alternative splicing perspective in particular.

## Materials and methods

### Genomic data

Three teleosts with high quality genomes and RNA-seq data were used in this study, zebrafish, medaka, and stickleback. Genomic data was retrieved from Ensembl (release 76). The RNA-seq data was retrieved from EBI, including 12 distinct tissues (brain, gills, heart, muscle, liver, kidney, bones, intestine, embryos, unfertilized eggs, ovary, and testis) in zebrafish; 11 tissues (brain, gills, heart, muscle, liver, kidney, bones, intestine, embryos, ovary, and testis) in medaka, and nine tissues (brain, gills, heart, muscle, liver, kidney, eye, skin, and testis) in stickleback (Additional file 1: Table S4).

### Alternative splicing form characterization

First, the transcript number of each gene in each of the three teleost species in Ensembl was obtained with BioMart [43] (Additional file 1: Table S1). Then, alternative splicing forms for each gene was predicted with RNA-seq data using the R package of SGSeq [44], as briefly described below. SGSeq provides an algorithm for prediction and quantification of alternative splicing forms from RNA-seq data and enables identification of unannotated and complex splice events, in which splice junctions and exons are predicted from reads mapped to the reference genome. High quality RNA-seq reads from different tissues in each species (Additional file 1: Table S4) were aligned to reference genome using HISAT2-2.1.0 [45] with option ‘--dta-cufflinks’. Resulting SAM files were subsequently sorted, merged, and filtered using SAMtools version 1.8 [46], e.g., only properly paired reads being retained. As such, RAN-seq data covered 98.8% of exon sites in zebrafish with mean coverage depth of 584.2, 98.7% of exon sites in medaka with mean coverage depth of 508.4, and 98.6% of exon sites in stickleback with mean coverage depth of 402.0 (Additional file 5: Fig. S4). In order to obtain the number of alternative splicing forms for each gene by SGSeq, BAM file for each gene in each of the three teleost species was extracted according to their position in genome. Then alternative splicing forms were predicted use the BAM file following SGSEq. Predicted alternative splicing form was further filtered according to gene annotation to ensure it was on the strand where gene was.

To test if occurrence of alternative splicing forms was equal between ohnologs, an exact binomial test was performed for predicted alternative splicing forms in each pair of ohnologs and resulting *P* values were corrected with Benjamini-Hochberg method [47] at a false discovery rate (FDR) threshold of 0.05.

### Gene Ontology enrichment

GO terms of each gene in the three teleost species were obtained with BioMart. GO enrichment analysis was performed to test whether ohnologs with asymmetric alternative splicing forms were significantly enriched certain GO terms with the R package of clusterProfiler [48]. For ohnologs with asymmetric alternative splicing forms in zebrafish, a GO-like enrichment of anatomical terms analysis was performed using the R package of BgeeDB [49, 50] to test if those ohnologs were preferentially expression in certain tissues by comparing to all ohnologs.

## Supplementary Information

**Additional file 1: Table S1.** Genes used in this study, and their transcript numbers in Ensembl and alternative splicing forms based on RAN-seq data. Details of gene identification and selection could be found in Inoue et al. [33] and Guo [21]. **Table S2.** The number of ohnolog pairs and their singleton orthologs. **Table S3.** GO-like enrichment of anatomical terms analysis of ohologs with significantly asymmetric splicing forms in zebrafish using BgeeDB (https://bgee.org/). **Table S4.** Information of RNA-seq data used in this study.

**Additional file 2: Fig. S1.** Transcripts number between ohnologs and their singleton orthologs. The number on the top of the box is the mean of each group.

**Additional file 3: Fig. S2.** Distribution of alternative splicing forms in singletons and ohnologs based on prediction with RNA-seq data.

**Additional file 4: Fig. S3.** Alternative splicing forms between ohnologs and their singleton orthologs from RNA-seq data in liver. The number on the top of the box is the mean of each group.

**Additional file 5: Fig. S4.** Distribution of coverage depth per exon site.

## Data Availability

All data generated or analysed during this study are included in this published article and its Additional files.

## Abbreviations

WGD: Whole genome duplication
TGD: Teleost genome duplication
FDR: False discovery rate
GO: Gene ontology
