# Supplementary figures and images for: The divergence of alternative splicing between ohnologs in teleost fishes
Source: BMC Ecol Evol. 2021 May 25;21:98. doi: 10.1186/s12862-021-01833-6 (PMC8146666; doi:10.1186/s12862-021-01833-6)

Zeb\_Med

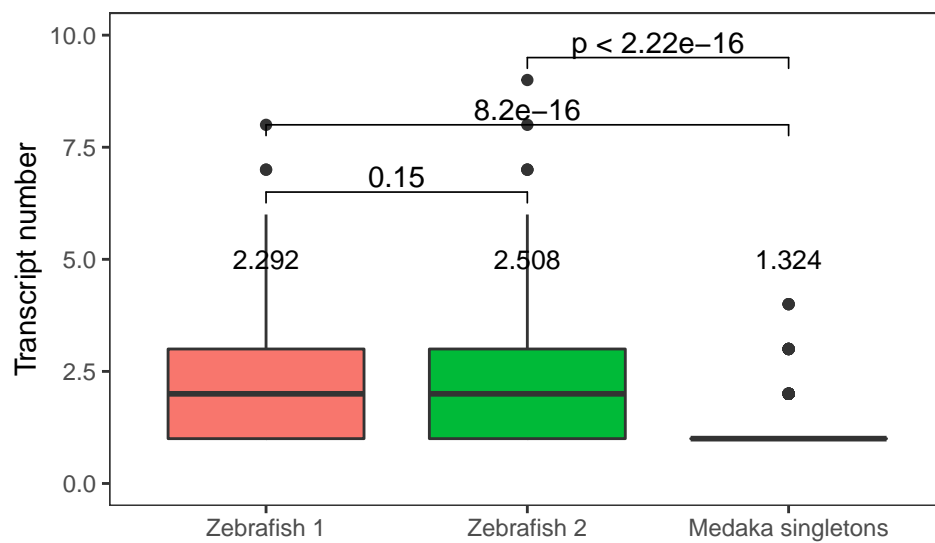

Zeb\_Sti

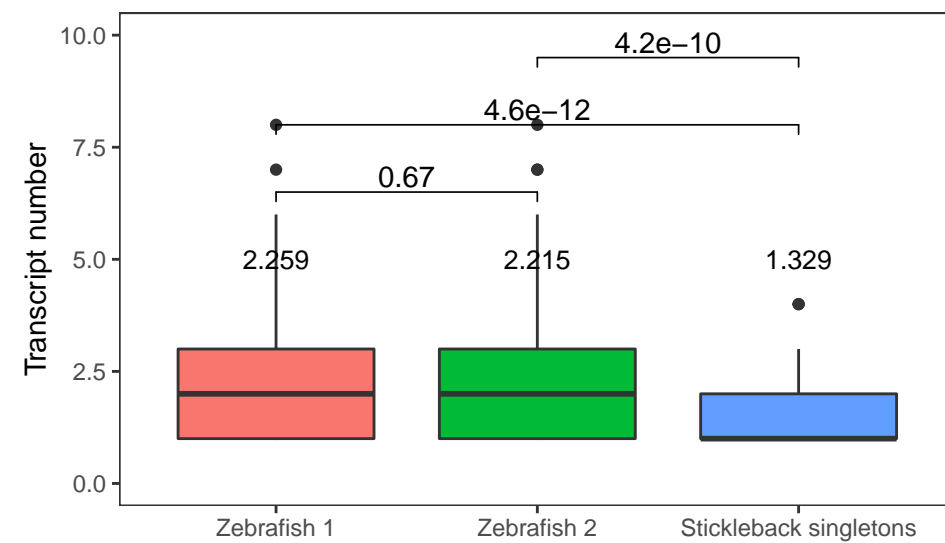

Med\_Zeb

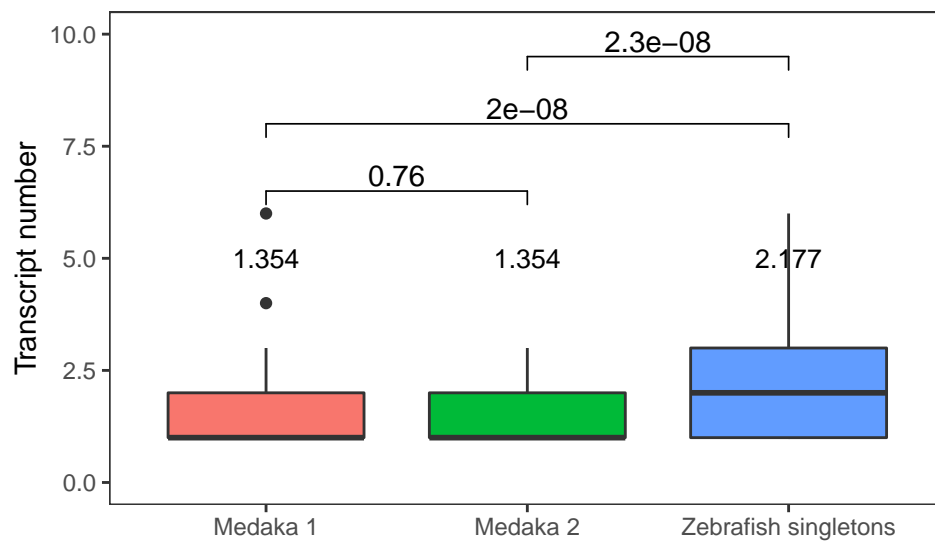

Med\_Sti

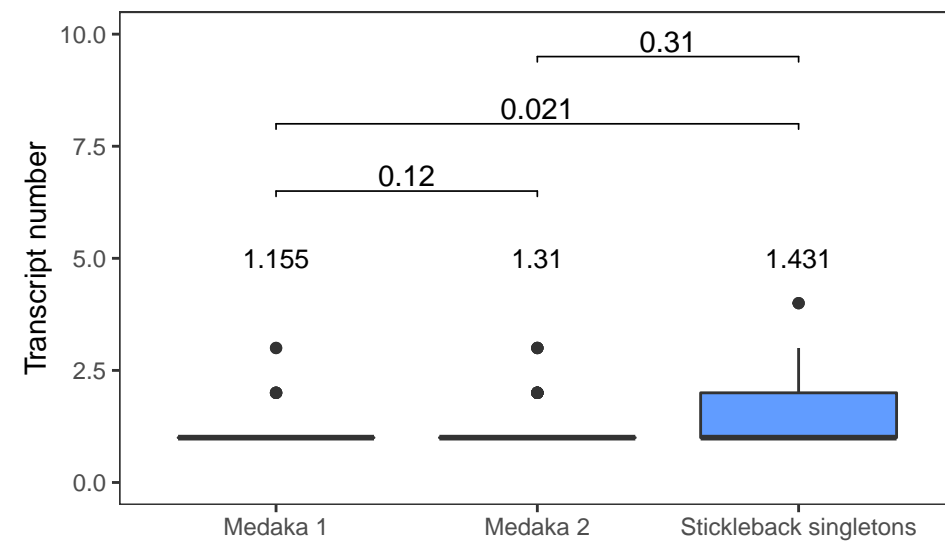

Sti\_Zeb

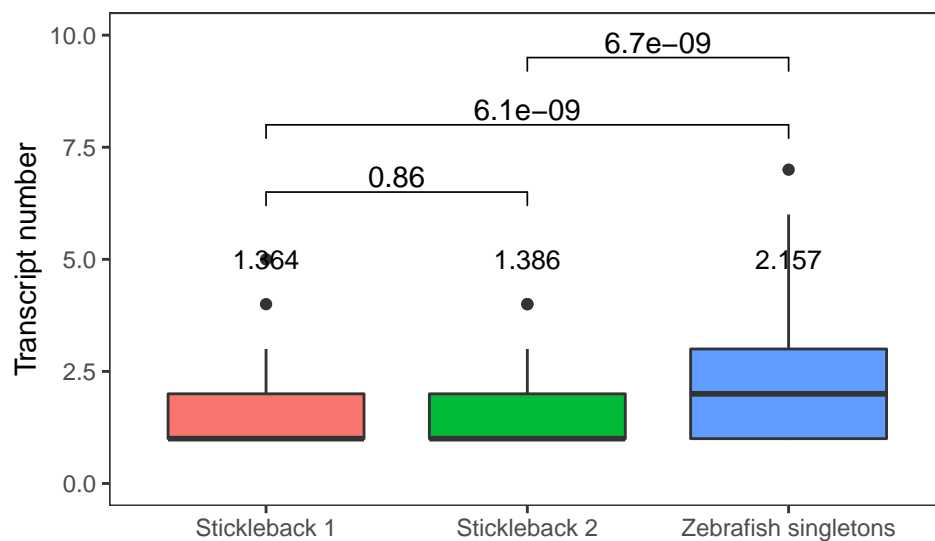

Sti\_Med

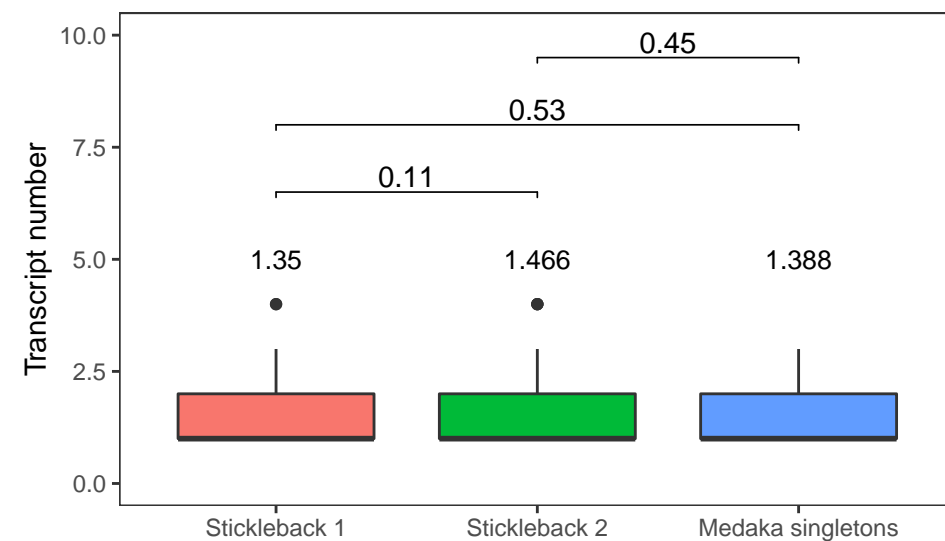

Supplement: Supplementary file 2 — Additional file 2: Fig. S1. Transcripts number between ohnologs and their singleton orthologs. The number on the top of the box is the mean of each group. [file 12862_2021_1833_MOESM2_ESM.pdf]

Zebrafish

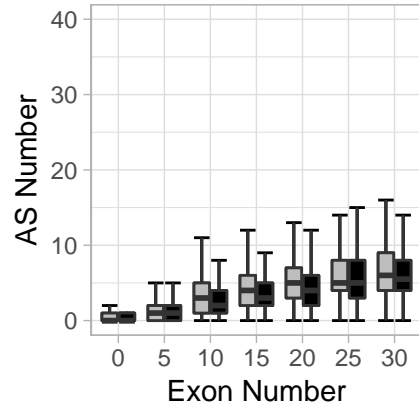

Medaka

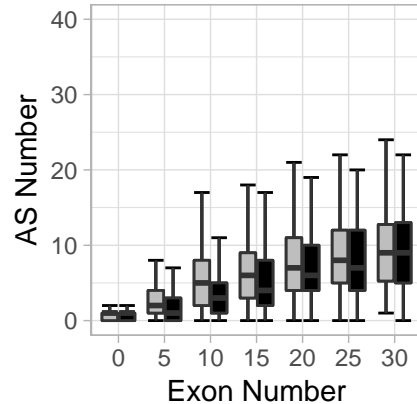

Stickleback

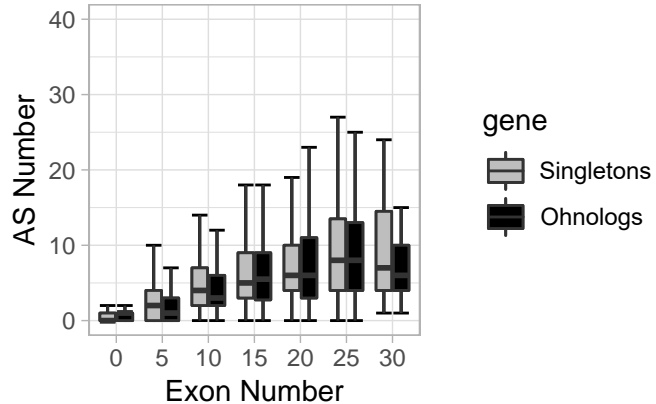

Supplement: Supplementary file 3 — Additional file 3: Fig. S2. Distribution of alternative splicing forms in singletons and ohnologs based on prediction with RNA-seq data. [file 12862_2021_1833_MOESM3_ESM.pdf]

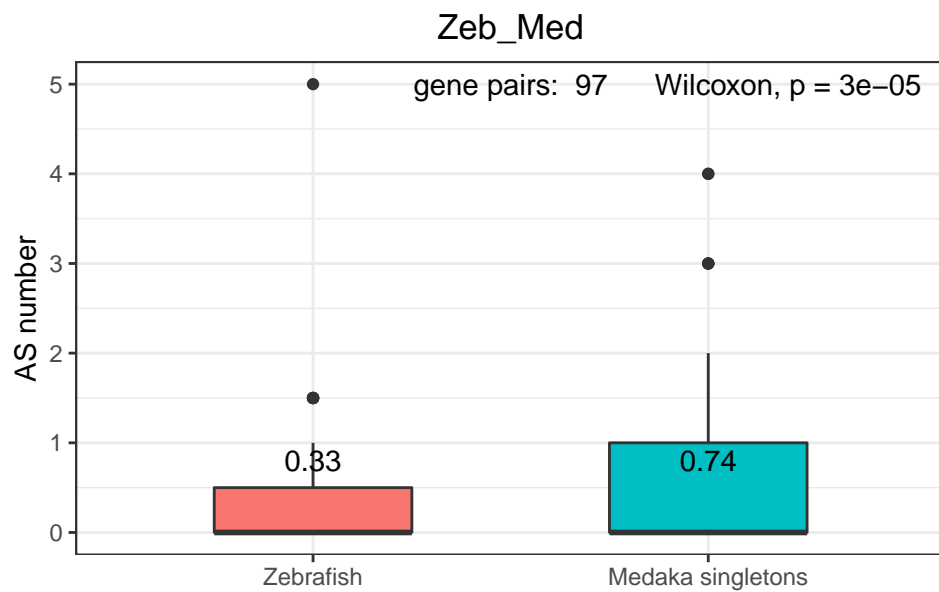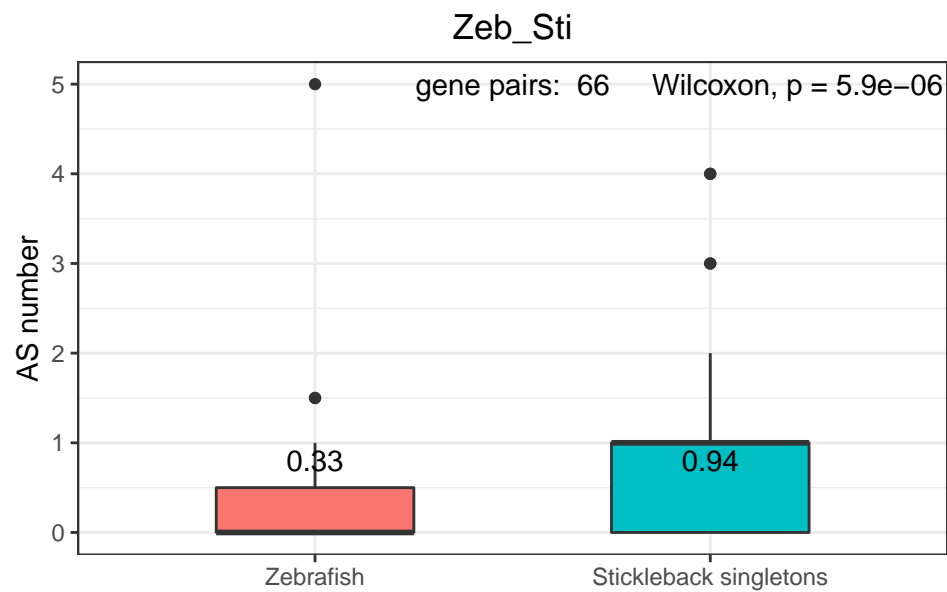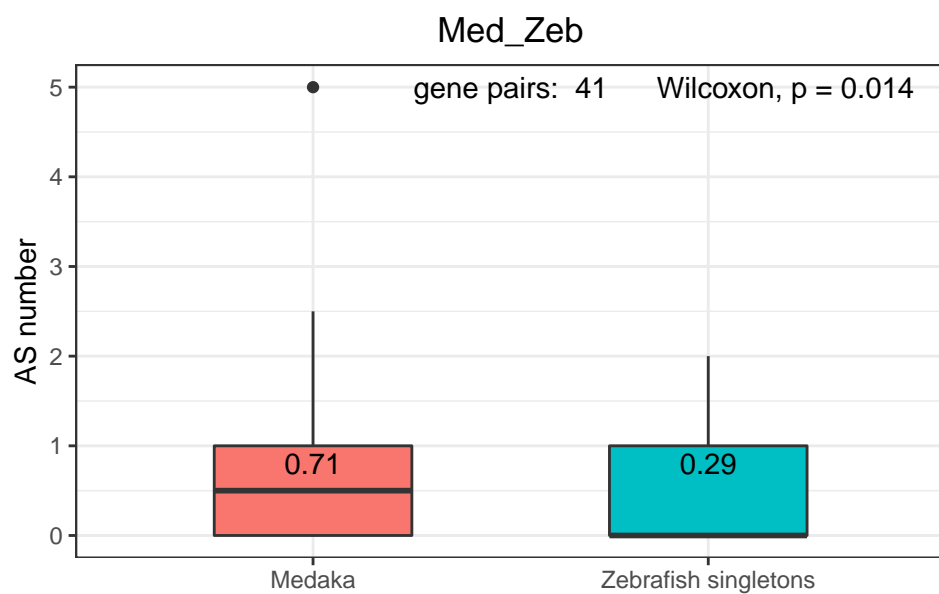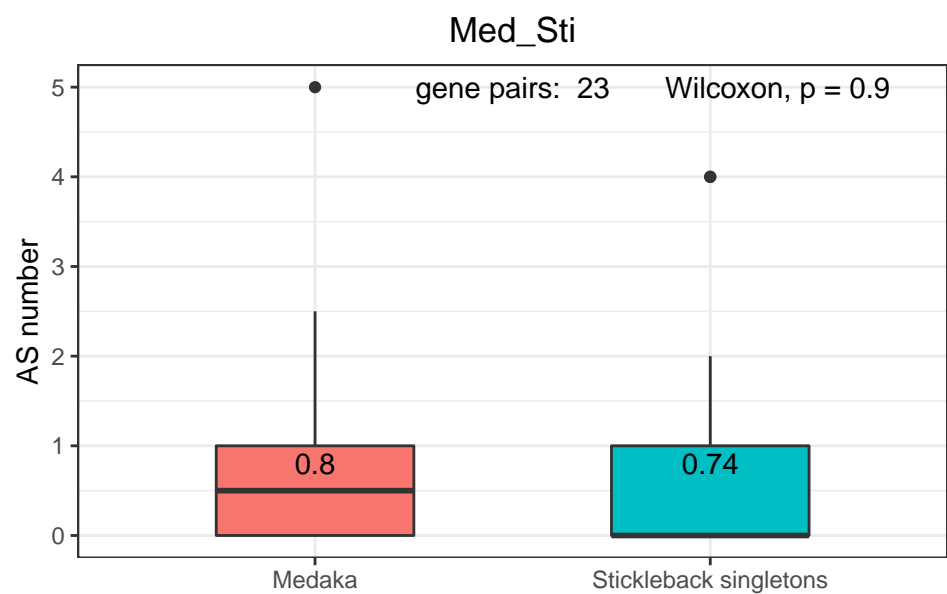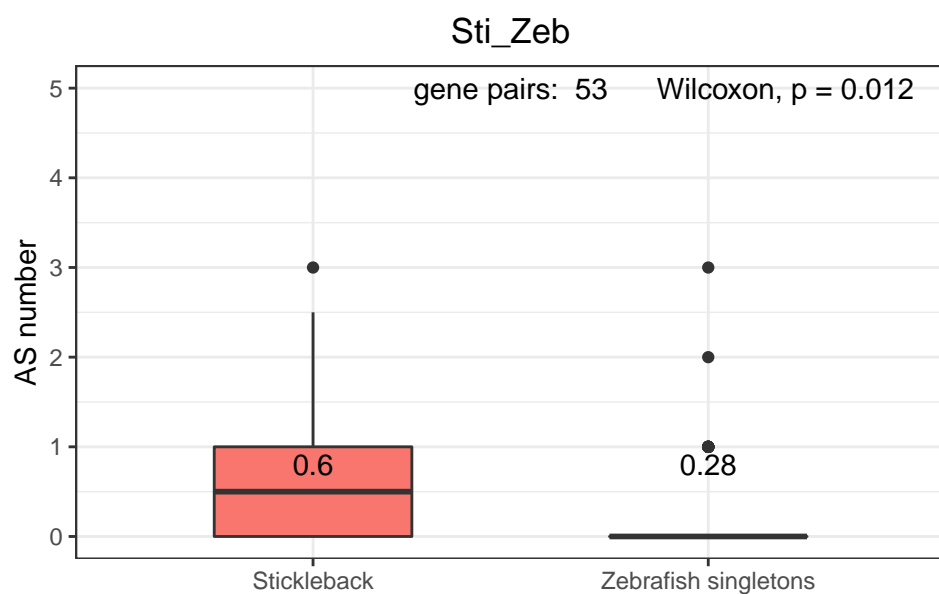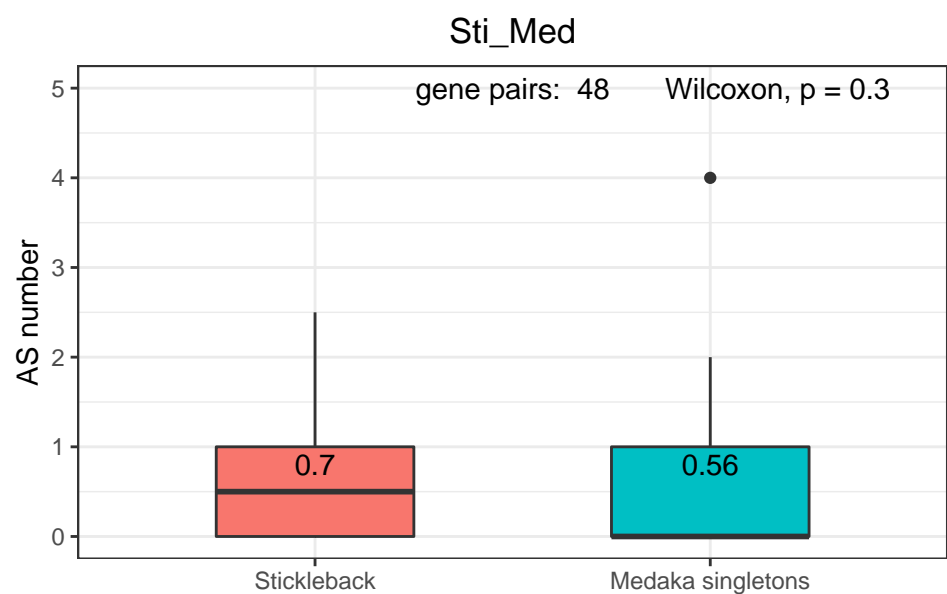

Supplement: Supplementary file 4 — Additional file 4: Fig. S3. Alternative splicing forms between ohnologs and their singleton orthologs from RNA-seq data in liver. The number on the top of the box is the mean of each group. [file 12862_2021_1833_MOESM4_ESM.pdf]

### Zebrafish

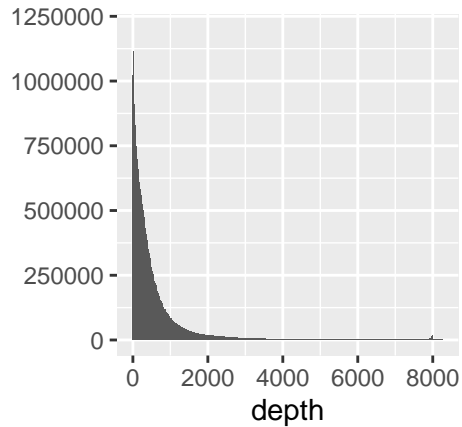

### Medaka

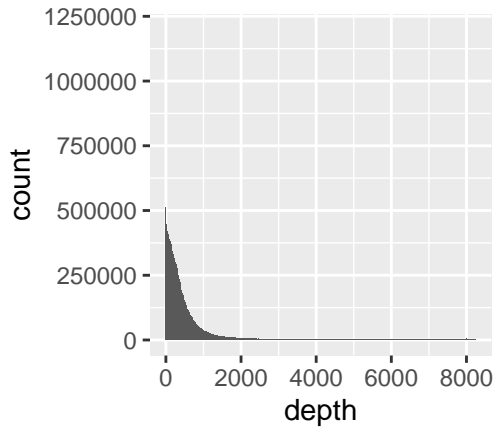

### Stickleback

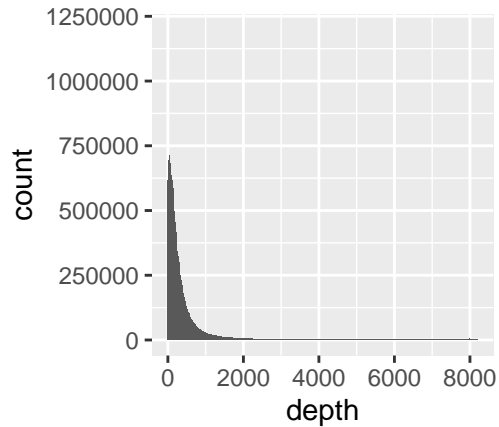

Supplement: Supplementary file 5 — Additional file 5: Fig. S4. Distribution of coverage depth per exon site. [file 12862_2021_1833_MOESM5_ESM.pdf]
